# Activity and Function of the PRMT8 Protein Arginine Methyltransferase in Neurons

**DOI:** 10.3390/life11111132

**Published:** 2021-10-24

**Authors:** Rui Dong, Xuejun Li, Kwok-On Lai

**Affiliations:** Department of Neuroscience, City University of Hong Kong, Hong Kong, China; ruidong@cityu.edu.hk (R.D.); xuejunli2@cityu.edu.hk (X.L.)

**Keywords:** neuron, synapse, dendritic spine, actin cytoskeleton, GTPase, post-translational modification

## Abstract

Among the nine mammalian protein arginine methyltransferases (PRMTs), PRMT8 is unusual because it has restricted expression in the nervous system and is the only membrane-bound PRMT. Emerging studies have demonstrated that this enzyme plays multifaceted roles in diverse processes in neurons. Here we will summarize the unique structural features of PRMT8 and describe how it participates in various neuronal functions such as dendritic growth, synapse maturation, and synaptic plasticity. Recent evidence suggesting the potential role of PRMT8 function in neurological diseases will also be discussed.

## 1. Introduction

Arginine methylation is a prominent protein post-translational modification identified decades ago [1]. Despite being detected in the brain, the function of arginine methylation is not generally well studied in neurons. In recent years, however, an increasing number of studies have unraveled how protein arginine methyltransferases (PRMTs), the enzymes which catalyze arginine, are involved in neuronal function. In particular, our understanding of arginine methylation has increased since the discovery of PRMT8 in 2015. PRMT8 was identified from the expressed sequence tag (EST) databases based on conserved motifs present in PRMTs [2]. It belongs to the type I PRMTs and shares almost 80% amino acid sequence identity with PRMT1. However, unlike PRMT1, which is ubiquitously expressed, the expression of PRMT8 appears to be exclusive in the brain [2]. Besides this unusual tissue distribution, PRMT8 also displays two unique properties: its ability to anchor to the plasma membrane and the presence of not only methyltransferase activity but also the catalytic activity of phospholipase.

## 2. PRMT8 Characteristics

### 2.1. Domain and Structure

The PRMT family of enzymes consists of nine members, which are classified into three types (I, II, and III) based on their catalytic activities. Type I PRMTs (PRMT1, 2, 3, 4, 6, and 8) catalyze the formation of both asymmetric dimethylarginine (ADMA) and monomethylated arginine (MMA); type II PRMTs (PRMT5 and 9) catalyze symmetric dimethylarginine (SDMA) and monomethylated arginine (MMA) formation; type III PRMT (PRMT7) only catalyzes monomethylated arginine (MMA) formation [3].

**Figure 1:**
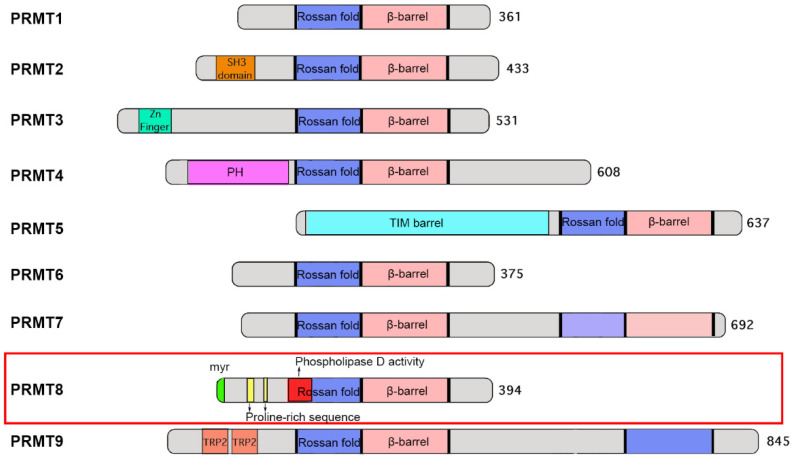
Schematic diagram illustrating the domain architecture of various PRMTs.

The canonical PRMT core structure adopts a conserved Rossman fold domain followed by a β-barrel domain where the dimerization arm is located [4]. Most PRMTs contain one catalytic Rossman fold domain, but dimerization through the β-barrel is required to compose the active enzyme. While the catalytic core domain of all PRMTs is structurally conserved, the N-terminal non-catalytic domain is very diverse among family members [5]. For example, PRMT3 contains a zinc finger motif which is required for its interaction with the ribosomal protein rpS2 to recognize RNA-associated substrates [6,7]; in contrast, PRMT5 contains a TIM barrel which is responsible for its interaction with the WD40 repeat protein MEP50 [8,9]. For PRMT8, there are a number of unique structural features in the N-terminal half of the protein (Figure 1): first, there is the presence of a myristoylation site at the N-terminus, which mediates its anchorage to the plasma membrane [2]. Second, phospholipase activity is present within the Rossman fold, making it the only PRMT that contains dual enzyme activities of methyltransferase and phospholipase [10]. These two distinct enzyme activities contribute to different functions of PRMT8 in neurons (see below). Third, its N-terminal region harbours two proline-rich sequences which allow its binding to SH3 domain-containing proteins such as Fyn (a protein tyrosine kinase), p85 (a regulatory subunit of PI3K) and PRMT2 [11]. The functional significance of the interactions between PRMT8 and these SH3 domain-containing proteins remains to be determined.

### 2.2. Expression Profile and Subcellular Localization

In contrast to the ubiquitous expression of most PRMTs, PRMT8 has restricted tissue distribution and is specifically expressed in the neurons of the central nervous system (CNS) [10]. PRMT8 is expressed in multiple brain areas such as the cerebral cortex, hippocampus and cerebellum [12]. At the subcellular level, PRMT8 can be localized to the plasma membrane through its unique N-terminal myristoylation motif [2,11]. The N-terminal 20 amino acids of PRMT8 are responsible for plasma membrane targeting by combining its myristoylation with the basic amino acids, and oligomerization/dimerization of PRMT8 enhances the membrane localization [13]. Interestingly, PRMT8 is also present at neuronal synapses [14,15], which are the specialized cellular compartments where neurotransmission between individual neurons takes place. Current evidence suggests that the local methylation of substrates by PRMT8 near the synapse is crucial for the development and plasticity of neuronal connections, which are pivotal to cognitive functions of the brain (see below).

While studies have mostly focused on the neuronal function of PRMT8 in the brain, PRMT8 might be important in glial cells as well, especially in the formation of glioblastoma. Reduced transcript expression PRMT8 is observed in glioblastoma patient tissues, suggesting its downregulation during tumor development in the brain [16]. Moreover, PRMT8 depletion in mouse embryonic stem cells (ESCs) has increased the expression of cellular markers which are associated with gliomagenesis [17]. However, the mechanism underlying PRMT8 in the pathogenesis of glioblastoma is still unclear. Intriguingly, somatic mutations and altered expression of the *PRMT8* gene have been found in cancer cells outside the brain [18,19,20]. The non-neuronal function of PRMT8, especially in the context of cancer progression, warrants further investigation.

## 3. Enzymatic Properties of PRMT8

### 3.1. Regulation and Crosstalk with Other PRMTs

The methyltransferase activity of PRMTs can be regulated in multiple ways. For example, PRMT activity can be regulated by auto-methylation. An auto-inhibitory mechanism for PRMT1, 4, 6, and 8 has been described [11,21]. For PRMT8, its enzymatic activity is negatively regulated by the first 60 amino acid residues at the N terminus [11], which contains two auto-methylation sites. It has been proposed that upon methylation of its substrates, the availability of fewer unmethylated substrates increases PRMT8 auto-methylation. This causes its N-terminus to bind to the enzyme core and prevents the methyl donor AdoMet from entering the catalytic site, thereby reducing PRMT8 activity [22]. The activity of PRMT can also be modulated through interacting with regulatory proteins that can either activate, inhibit, or change the specificity of PRMTs substrates [23,24]. In this regard, it is noteworthy that PRMT8 binds to Fyn and p85 through its proline-rich sequences in the N-terminal region [11]. Given that the N-terminal of PRMT8 is involved in its own auto-inhibition, the binding of these proteins to the N-terminal region may release the auto-inhibition of PRMT8 [11], a possibility that requires further investigation.

Two other PRMTs, namely PRMT1 and PRMT2, have been found to interact with PRMT8. The formation of heterodimers between PRMT8 with PRMT1 may recruit PRMT1 activity to the plasma membrane [2]. PRMT8 can also interact with the SH3 domain of PRMT2 through the two proline-rich sequences in the N-terminal region [11]. However, the precise function of the interaction or cross-talk between PRMT8 and other family members remains unclear.

There are pharmacological inhibitors of the PRMT family of enzymes. These include simple SAM analogs sinefungin, SAH, methylthioadenosine (MTA), and AzaAdoMet, which are often used as tools to change the global methylation levels in cells [25]. However, these are Pan-MTase inhibitors and do not target specific PRMT family members. Up to now, specific inhibitors have been developed for PRMT1, PRMT3, PRMT4, PRMT5, PRMT6, and PRMT7 [3,25]. Although several compounds can inhibit PRMT8, their action also extends to other PRMTs such as PRMT1, PRMT4, PRMT5, and PRMT6 [25]. Therefore, a specific PRMT8 inhibitor has yet to be identified.

### 3.2. Substrates of PRMT8

As PRMT8 is structurally most similar to PRMT1, they may share similar catalytic activities and substrate specificities. Both enzymes can methylate the same substrates, such as Npl3, GAR, and histone H4, in vitro [2]. Nonetheless, relatively few substrates of PRMT8 have been identified.

The pro-oncoprotein Ewing sarcoma protein (EWS) has been reported to be a PRMT8 substrate. The interaction between EWS and PRMT8 is mediated through the C-terminal RGG3 domain of EWS and the AdoMet binding domain of PRMT8 in the N-terminal region [26,27,28,29]. However, the precise role of methylation by PRMT8 towards regulating EWS function remains unknown. The nucleolar protein interacting with the fork head-associated (FHA) domain of Ki-67 (NIFK), a RNA binding protein, has also been demonstrated to act as a PRMT8 substrate by in vitro methylation, and methylation of NIFK is required for large subunit ribosomal RNA maturation [30].

Voltage-gated sodium channel (Nav), which functions to initiate action potentials in neurons, contains methylation sites and is a putative PRMT8 substrate. Although the PRMT that methylates Nav in vivo has not been identified, co-expression of the major brain Nav channel Nav1.2 with PRMT8 causes a striking 3-fold increase in the Nav1.2 current [31], indicating Nav1.2 as a potential candidate of PRMT8 substrate. Notably, increased methylation of Nav1.2 has been detected in mouse brain after acute seizures induced by kainic acid. As a consequence, sodium channel function is altered, which affects neuronal excitability. It would be important to investigate in the future whether alteration of PRMT8 expression or activity and the subsequent changes in arginine methylation of sodium channels may occur in epileptic patients.

The RasGAP SH3 domain-binding protein 1 (G3BP1), an RNA-binding protein crucial for the formation of stress granules that limit protein synthesis during cellular stress, such as oxidative stress, ultraviolet radiation and viral infection, is a substrate of PRMT8 [15,32]. In non-neuronal cells, the arginine methylation of G3BP1 prevents the assembly of stress granules in response to oxidative stress [32]. On the other hand, the effect of PRMT8 in synapse maturation of neurons is mediated, at least in part, through the methylation of G3BP1 and modulation of synaptic actin dynamics [15].

## 4. Neuronal Functions of PRMT8

### 4.1. PRMT8 Functions as a Phospholipase to Regulate Purkinje Cell Dendritic Arborization

Among the different PRMTs, PRMT8 is the only family member that possesses both methyltransferase and phospholipase activities. The phospholipase activity of PRMT8 directly catalyzes the hydrolysis of phosphatidylcholine (PC) to generate choline and phosphatidic acid (PA) [10]. PC is a major component of biological membranes and is dynamically regulated through hydrolysis and biosynthesis [33,34]. The metabolism of PC is highly involved in multiple morphogenetic processes during neuron development, such as axonal outgrowth, elongation, and neurite branching [35,36]. PC modulates neuronal functions from two aspects. First, PC-hydrolyzed choline is converted to acetylcholine, a major neurotransmitter that regulates multiple brain functions and animal behaviors [37]. Second, phosphatidic acid modulates morphological changes in neurons through its action on cytoskeleton remodeling and plasma membrane rearrangement [38]. Like other eukaryotic phospholipase D (PLDs), PRMT8 has a typical catalytic HxKxxxxD (HKD) motif which is unique among all PRMTs, suggesting the role of PRMT8 as a phospholipase [39,40]. Indeed, aberrant reduction of acetylcholine and choline levels, as well as increased PC levels, are detected in the cerebellum of *Prmt8^−/−^* mice [10]. Using MALDI-QIT-TOF/MS, Kim et al. further identify lysine-107 on PRMT8 as the essential amino acid residue for its phospholipase activity in vitro, and its PC hydrolysis activity promotes neurite branching in PC12 cells upon treatment with nerve growth factor (NGF) [10,41].

Purkinje cells located in the cerebellar cortex have highly elaborate dendritic trees, whose morphological changes are closely related to animal motor performance [42]. Purkinje cells are well known for their critical role in maintaining cerebellar functions including motor coordination and attention [42]. Dendritic arborization of Purkinje cells is regulated by multiple signaling processes including external cues and internal molecular pathways such as PC metabolism [43,44]. In the mouse brain, PRMT8 has predominant expression in the descending axons and dendritic arbors of Purkinje cells along development [12]. In *Prmt8* homozygous knockout mice, Purkinje cells display stunted dendritic trees and reduced dendritic arborization [10]. Moreover, *Prmt8^−/−^* mice exhibit increased spontaneous behavioral hyperactivity and gait abnormalities, supporting an essential role of PRMT8 in cerebellar-related functions [10]. Numerous studies have linked PLD activity to brain development and functions. PLD-deficient mice have delayed brain growth and impaired cognitive functions [45]. Moreover, PLDs are implicated in Alzheimer’s disease (AD) with reduced catalytic activities in neurons carrying familial Alzheimer’s disease-related PS1 mutation [46]. Given the fact that PRMT8 has dual enzymatic activities of phospholipase and arginine methyltransferase, further studies into the crosstalk between the dual catalytic activities of PRMT8 will provide more information on how PRMT8 regulates neuronal functions and its relationship to neurological diseases [10,47].

### 4.2. PRMT8 Regulates Synaptic Plasticity and Cognitive Functions

Our brain function relies on communication between neurons through neurotransmission at specific junctions called synapses. Among all the PRMTs, PRMT8 is one of the few that show relatively enriched expression in the mouse synaptoneurosome, a biochemical preparation of synaptic components from the brain [14,15]. Upon N-terminal myristylation, PRMT8 can be membrane-bound [2,4], indicating the possibility that it may be targeted to the synapse. Based on these unique properties, PRMT8 might represent an important regulator in synaptic function. Indeed, Penny et al. have demonstrated that PRMT8 is localized in both pre- and post-synaptic compartments of cultured neurons, whereas relatively little PRMT8 signal is detected in the nuclear fraction of the brain [14]. Similar observations on the synaptic localization and low abundance in the nucleus was confirmed in a later study [15], suggesting the potential role of PRMT8 outside the nucleus, especially at the synapse. In mice with selective deletion of *Prmt8* in nestin-expressing cells, aberrant synaptic plasticity with increased miniature excitatory postsynaptic currents (mEPSC) frequency and amplitude in hippocampal slices has been observed, while no significant difference was detected in the miniature inhibitory postsynaptic currents (mIPSC), suggesting a specific role of PRMT8 in excitatory synapse function. Furthermore, the expression of several synaptic proteins, such as the NMDA receptor subunit GluN2A and eukaryotic translation initiation complex (eIF4E, eIF4G1, and eIF4H), is reduced in *Prmt8* conditional knockout (cKO) brain lysates and synaptosome, while their mRNA levels were not altered [14]. Thus, consistent with its prominent expression outside the neuronal nucleus, PRMT8 can regulate synaptic function through a post-transcriptional mechanism. In the rodent hippocampus, a brain area important for spatial memory, NMDAR is crucial for synaptic plasticity, which is a major cellular mechanism that underlies learning and memory [48,49]. GluN2A is one of the most common NMDAR regulatory subunits and GluN2A-containing NMDARs are enriched in the postsynaptic density (PSD) [50,51,52]. GluN2A is important in cognitive functions including contextual fear memory formation and spatial working memory [53,54,55]. Consistent with the altered level of GluN2A, the GluN2A-mediated NMDAR currents are reduced in hippocampal slices from *Prmt8* cKO mice. Consequently, the *Prmt8* cKO mice exhibit impaired contextual fear memory which might be due to the altered synaptic functions [14]. How PRMT8 affects expression of GluN2A and other synaptic proteins at the post-transcriptional level and in turn regulates synaptic function remains to be explored.

### 4.3. PRMT8 Regulates the Maturation of Synapse and Neural Circuit during Brain Development

In addition to the hippocampus, PRMT8 has been reported to influence the function of interneurons in the developing visual cortex. *Prmt8* ablation disrupts the proteome related to axonal and dendritic development, which might account for the reduced visual acuity and increased parvalbumin neuron complexity [56]. In this study, PRMT8 was found to modulate the transcription of gene encoding Tenascin-R, a component in the peri-neuronal net, thereby influencing dendritic arborization and synaptic functions.

Protein arginine methylation has well-documented roles in the nucleus, including gene transcription, RNA splicing, RNA export, and chromatin remodeling [57,58,59]. On the other hand, few studies have explored its significance in neurons outside the nucleus. The *Prmt8* mRNA is among the ~2000 transcripts present in the hippocampal neuropil of mouse brain [60], supporting a possible function of locally synthesized PRMT8 in a cellular compartment such as neuronal synapse. Interestingly, PRMT8 expression is substantially upregulated during postnatal days 7 to 21 in the mouse hippocampus [15], which correlates with the maturation of dendritic spines [61]. Dendritic spines are small protrusions on dendrites where most excitatory synapses locate. Dendritic spines are classified based on their morphologies. In general, the elongated filopodia which are prominent in the developing neurons are motile and transient, while the mushroom spines with large heads are more stable and involved in memory consolidation [62]. Filopodia density reaches the peak at an early development stage and then begins to decline, with more mushroom spines being formed for synapse maturation. Loss of PRMT8 after introduction of short-hairpin RNA (shRNA) results in the overproduction of filopodia. Increased filopodia density is also observed in heterozygous or homozygous *Prmt8* knockout neuron [15]. Several studies have reported the association of defective spine maturation with abnormal animal behaviors [63,64,65]. Indeed, the *Prmt8*-deficient mice display altered anxiety levels in open field test and elevated plus maze test, while sociability is not affected [15]. Thus, PRMT8 is an important regulator of dendritic spine maturation, and PRMT8 deficiency results in selective abnormal animal behaviors.

PRTM8 is present at excitatory synapses and dendritic spines of cultured hippocampal neurons, and it promotes spine maturation through its arginine methyltransferase activity instead of phospholipase activity. Notably, the spine defects caused by the PRMT8 deficiency cannot be rescued by the nuclear-restricted PRMT8, indicating that PRMT8 acts outside the nucleus to promote dendritic spine maturation [15]. Dendritic spines are actin-enriched protrusions and their morphology is tightly controlled by remodeling of the actin cytoskeleton [66,67]. PRMT8 suppresses filopodia formation via the control of Rac1-PAK signaling, which regulates actin dynamics through phosphorylation of the actin-depolymerization factor cofilin. Furthermore, G3BP1, a dendritic RNA-binding protein, has been identified as the downstream substrate of PRMT8 in dendritic spine maturation [15]. G3BP1 is an essential component of stress granules, but little is known about the neuronal function of G3BP1 under normal physiological conditions in the absence of cellular stress [68]. Hippocampal neurons of *G3bp1* knockout mice show exaggerated long-term depression (LTD), indicating the crucial role of G3BP1 in synaptic plasticity [69]. Consistent with this notion, we found that G3BP1 is essential for dendritic spine maturation and actin remodeling, and these functions depend on arginine methylation within the RGG domain of G3BP1 [15]. The precise mechanism by which PRMT8 and G3BP1 regulate Rac1-PAK signaling requires further investigation. However, enhanced eIF4G in the translation initiation complex is observed in the brain upon *Prmt8* deficiency [15]. Since an increase in eIF4E-eIF4G interaction and subsequent elevation of protein synthesis can hyperactivate the Rac1-PAK signaling and impair dendritic spine maturation [70], it is tempting to speculate that the alteration of protein synthesis may contribute to defective actin dynamics in PRMT8- or G3BP1-depleted neurons.

**Figure 2:**
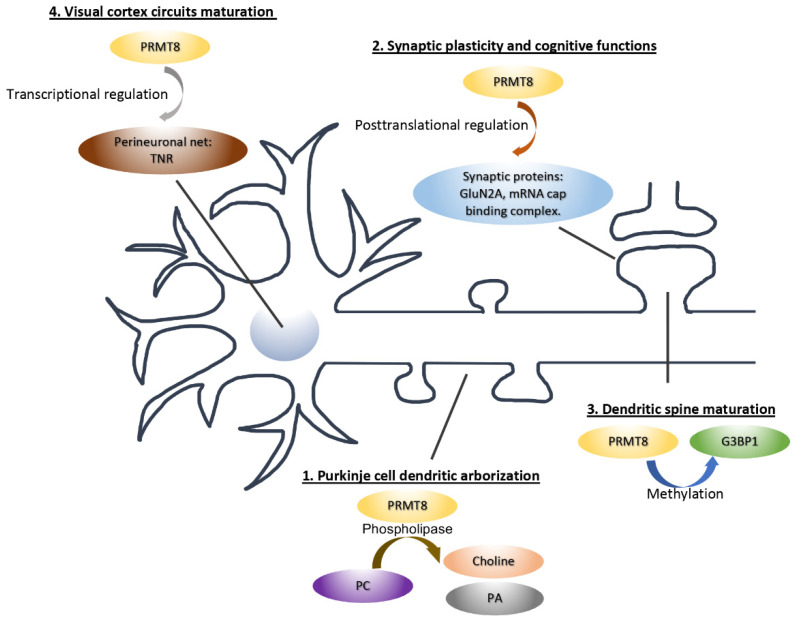
The cellular functions and molecular mechanisms of PRMT8 in neurons. PRMT8 can function both inside and outside the nucleus. The mechanisms of PRMT8 include (1) the production of choline and phosphatidic acid (PA) that promotes dendritic growth; (2) transcription in the nucleus to regulate perineuronal net expression; (3) the regulation of actin dynamics through the RNA-binding protein G3BP1; and (4) the synthesis of synaptic proteins essential for synaptic plasticity and memory formation.

A large number of arginine-methylated proteins have been discovered in the adult mouse brain by mass spectrometry. Notably, among the many putative substrates of PRMTs in the brain are synaptic proteins [71], implying that protein arginine methylation could represent a major post-translational modification in the regulation of synaptic function and PRMT8 may not be the only arginine methyltransferase involved. Indeed, emerging evidence supports the role of other PRMTs in dendritic spine formation and maturation. The coactivator-associated arginine methyltransferase 1 (CARM1/PRMT4) is present in the dendrite and is co-localized with the postsynaptic protein PSD-95. Loss of PRMT4 promotes mushroom spines formation in cultured hippocampal neurons, which is due to the increased number and size of PSD-95 and GluN2B subunits of NMDA receptor at the synapse [72]. On the other hand, PRMT3 modulates dendritic spine maturation in hippocampal neurons through the maintenance of BDNF-dependent local mRNA translation [73]. Furthermore, PRMT2 is crucial for the dendritic arborization of young neurons through the arginine methylation of Cobl, which is an actin nucleation factor [74]. Whether the same PRMT2-Cobl pathway in involved in dendritic spine maturation at later developmental stage remains to be determined. Altogether, these studies provide compelling evidence that multiple PRMTs are critical to neuronal development and synapse maturation that extend beyond their conventional functions as the regulators of transcription and RNA processing within the nucleus (Figure 2).

### 4.4. PRMT8 and Neurological Diseases

Several recent studies have suggested the link between PRMT8 and neurological diseases. One type of motor neuron disease, amyotrophic lateral sclerosis (ALS), has a pathological hallmark of aggregated inclusion bodies in motor neurons. The RNA-binding proteins FUS and TDP-43 are the major components of inclusion bodies in the brain from ALS patients [75]. Various mutations have been identified in TDP-43 and FUS in FUS-TDP- 43/ALS-FUS patients [76,77]. Remarkably, PRMT1 and PRMT8 are present in inclusion bodies of cultured COS-1 cells carrying ALS-linked FUS mutations. Furthermore, the FUS-positive inclusion bodies in motor neuron-derived (MN-1) cells and cells from ALS-FUS patient are reduced upon the inhibition of PRMT enzyme activity [78]. In contrast, in a *Drosophila* model carrying ALS-FUS mutations, depletion of endogenous arginine methyltransferase 1 (DART1), which is orthologous to human PRMT1 and PRMT8, enhanced the neurodegenerative phenotype introduced by FUS overexpression in fly eyes [78]. This study thus demonstrated the potential role of PRMT8 in ALS pathogenesis through the regulation of RNA-binding proteins. The importance of PRMT8 in motor neuron function has also been investigated in *Prmt8*-deficient mice. Aged mice (~15 months) lacking PRMT8 exhibit impaired muscle strength with weak limb clasping and muscle atrophy, which are attributed to the dysfunctions of motoneurons in the spinal cord [79]. Increasing DNA double-strand breaks and defective stress tolerance are also found in the motoneurons of *Prmt8*-deficient mice. The dysfunction of motoneurons is tightly related to neurodegenerative disorders [80]. Since PRMT8 influences motor behaviors through its phospholipase activity [10], PRMT8 might represent a potential target for drug discovery in delaying degeneration of motoneurons and treatment for MN-related neurological disorders.

In addition to MN-related degeneration disorders, given that PRMT8 modulates the visual cortical circuit as an epigenetic regulator [56], dendritic spine maturation as regulator of actin dynamics [15], and the control of context-dependent fear learning and anxiety behavior [14,15], it is possible that PRMT8 might also be involved in neurodevelopmental disorders. Indeed, a recent paper has reported that the *PRMT8* gene is located within a common deleted chromosome region from patients with microcephaly [81], suggesting its possible role in human brain development and its dysfunction in neurodevelopmental disorders.

## 5. Conclusions and Outlook

PRMT8 is unique among all PRMTs due to its brain-restricted expression, the membrane anchorage through myristoylation, and the dual enzymatic activities of methyltransferase and phospholipase. The importance of PRMT8 has now been demonstrated in diverse neuronal functions, including dendritic arborization, dendritic spine maturation, synaptic plasticity, motor performance, and visual acuity. Mass spectrometry has identified numerous cytoplasmic and membrane proteins being arginine-methylated in the brain [71]. These include proteins as diverse as ion channels on the plasma membrane [31] and molecular motors on microtubule [82]. However, relatively few studies have been carried out to elucidate the role of protein arginine methylation outside the nucleus. Therefore, recent studies on the non-nuclear role of PRMT8 in neurons have substantially extended our understanding of the mechanism of protein arginine methylation. Besides synapse development and function, other cellular processes in neurons, including axonal trafficking and dendritic branching, also depend on protein arginine methylation [74,83]. It is worthwhile to further characterize the function of PRMTs in neurons outside the nucleus in the future. In particular, given that PRMT8 can be attached to the plasma membrane, further identification and characterization of membrane proteins as PRMT8 substrates would be pivotal to gain deeper understanding on how this enzyme works in the brain.

Numerous RNA-binding proteins have been identified as arginine-methylated proteins in the cytoplasm [57,84]. Mutations and abnormal expression of RNA-binding proteins have been reported to be engaged in different neurological diseases, including FMRP, TAR DNA binding protein 43 (TDP-43), Hu proteins, and FUS [84,85,86]. Despite the observations that *Prmt8*-deficient mice exhibited selective abnormal behaviors in motor performance and anxiety test, the in vivo role of PRMT8 in brain function has not yet been well defined, and its precise linkage with diseases is largely unexplored. In addition to G3BP1, searching for other RNA-binding proteins that are present in dendrite and synapses as the downstream targets of PRMT8 would be critical for illuminating the multifaceted functions of PRMT8 in neurodegeneration and neurodevelopmental disorders in the future.
